# Europium oxide nanorod-reduced graphene oxide nanocomposites towards supercapacitors

**DOI:** 10.1039/c9ra11012g

**Published:** 2020-05-06

**Authors:** Parisa Aryanrad, Hamid Reza Naderi, Elmira Kohan, Mohammad Reza Ganjali, Masoud Baghernejad, Amin Shiralizadeh Dezfuli

**Affiliations:** Center of Excellence in Electrochemistry, Faculty of Chemistry, University of Tehran Tehran Iran; Novin Ebtekar Company, Exclusive Agent of Metrohm-Autolab, Dropsens Companies Tehran Iran; Ronash Technology Pars Company Tehran Iran; Biosensor Research Center, Endocrinology & Metabolism Molecular-Cellular Sciences Institute, Tehran University of Medical Sciences Tehran Iran; Helmholtz-Institute Münster, Forschungszentrum Jülich GmbH Corrensstraße 46 48149 Münster North Rhine-Westphalia Germany; Radiation Biology Research Center, Iran University of Medical Sciences (IUMS) Tehran Iran amindezfuli@outlook.com

## Abstract

Fast charge/discharge cycles are necessary for supercapacitors applied in vehicles including, buses, cars and elevators. Nanocomposites of graphene oxide with lanthanide oxides show better supercapacitive performance in comparison to any of them alone. Herein, Eu_2_O_3_ nanorods (EuNRs) were prepared through the hydrothermal method and anchored onto the surface of reduced graphene oxide (RGO) by utilizing a sonochemical procedure (in an ultrasonic bath) through a self-assembly methodology. The morphologies of EuNRs and EuNR-RGO were characterized by scanning electron microscopy (SEM), X-ray diffraction (XRD) and IR spectroscopy. Then, we used EuNRs and EuNR-RGO as electrode materials to investigate their supercapacitive behavior using cyclic voltammetry, galvanostatic charge–discharge, and electrochemical impedance spectroscopy techniques. In a 3.0 M KCl electrolyte and with a scan rate of 2 mV s^−1^, EuNR-RGO exhibited a specific capacity of 403 F g^−1^. Galvanostatic charge–discharge experiments demonstrated a specific capacity of 345.9 F g^−1^ at a current density of 2 A g^−1^. The synergy between RGO's flexibility and EuNR's high charge mobility caused these noticeable properties.

## Abstract 2

Fast charge/discharge cycles are necessary for supercapacitors applied in vehicles including, buses, cars and elevators.

## Graphical abstract

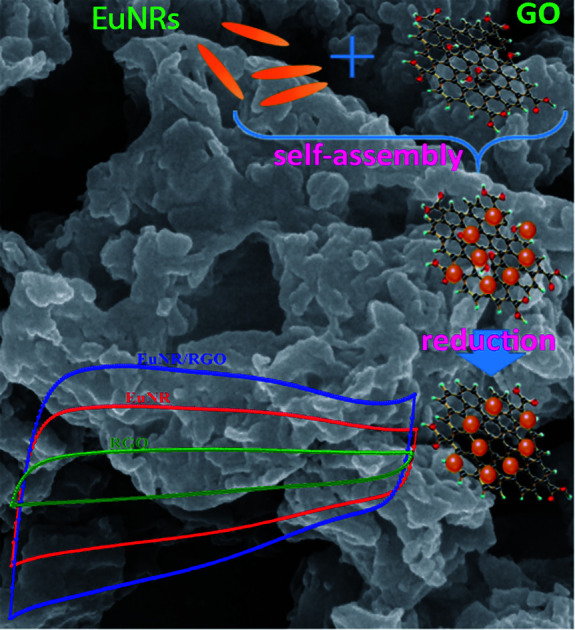

## 1. Introduction

Nowadays, supercapacitors due to their high energy density, have been investigated as storage devices and due to this property, they have been given particular priority over conventional dielectric capacitors. Because of some features such as good cycling stability, fast charge/discharge rate, and high power density, supercapacitors are superior to batteries.^1^ Electric double-layer capacitors (EDLCs) and pseudo-capacitors are major types of supercapacitors.^2–5^ In pseudo-capacitors, the electrodes contain oxides of noble and transition metals or conducting polymers, whereas the main materials in EDLCs are carbonaceous materials such as carbon nanotubes (CNTs) or graphene.^5,6^ The latter group of electrode materials owns unique features such as thermal and chemical stabilities, very high conductivity, and considerable surface area.^7^

Distinctive reduced graphene oxide (RGO), obtained through the reduction of graphene oxide (GO), owing to its low-cost approach has gained considerable attention among other nanomaterials.^8^ Moreover, reduced graphene oxide can be decorated with various nanomaterials and the properties of the resulting hybrid nanomaterials can be fine-tuned by modifying the loading degree as well as the type of the nanomaterial loaded on the RGO sheets.^8–13^ A group of materials used for the decoration of RGO comprises metal oxides like MnO_2_, Mn_3_O_4_, RuO_2_, CeO_2_, Yb_2_O_3_ and SnO_2_, which avoids decrease in the surface area of RGO *via* the interplanar spacing of metal oxides.^14,15^ Open places are formed among the sheets by anchoring metal oxides on RGO; thus, the internal resistance of the material is greatly diminished and subsequently, the penetration of electrolytes in the electrodes becomes smooth.^16^ Due to the above-mentioned modifications, the capacitance and energy density of carbonaceous supercapacitors have increased.^2,15,17,18^

On the other hand, numerous studies have been carried out on lanthanide oxides or in other words rare earth oxides (REOs), because of their extensive utilizations in fuel cells, heterogeneous catalysis, ion glass industries and electronics.^19–24^ Some REOs are considered potential candidates toward pseudo-capacitors due to their redox properties.^19,25^

Strategic rare earth compounds due to their electronic, optical and electrochemical characteristics arising from the electron transitions within the 4f shells, different conformation states and empty sites in the crystallization mode have been utilized in different fields such as high-performance luminescent devices, biochemical applications, catalysts and supercapacitors.^26^ Recently, the synthesis of one-dimensional Eu_2_O_3_ nanostructures has gained considerable attention since they possess higher packing density and a larger percentage of active sites in comparison with bulk materials.^27^ For instance, Pol *et al.*^28^ prepared Eu_2_O_3_ nanorods by the thermal transformation (700 °C) of ultra-sonication-induced Eu(OH)_3_ nanorods. In another study, Du *et al.*^29^ produced Eu_2_O_3_ nanorods through a chemical reaction at 90 °C by means of cyclohexylamine as the alkaline source. Wang *et al.*^30^ prepared Eu_2_O_3_ nanotubes, nanowires and nanorods by a hydrothermal approach. Short Eu_2_O_3_ nanorods were synthesized by Zhang *et al.*^31^ through a sol–gel method by using an aqueous solution of europium nitrate in the presence of ammonia and urea in a micro reactor made of polystyrene/polyelectrolyte. Qian *et al.*^32^ reported simple chemical precipitation for the synthesis of light rare earth hydroxide nanorods. However, a long aging time (30 days) was unavoidably needed.

In this work, we have introduced a facile two-step procedure for preparing Eu_2_O_3_ nanorods anchored on RGO (EuNR-RGO). The first step is the hydrothermal synthesis of the Eu_2_O_3_ nanorods (EuNRs) and the second step is a sonochemical self-assembly approach. By means of this process, the size of Eu_2_O_3_ decreased to the nanometer scale, due to which the surface area and redox activity were greatly enhanced. This enhancement caused special supercapacitive behavior.

## 2. Experimental

### 2.1. Materials

All the following reagents were utilized as purchased without further purification. Sulphuric acid (H_2_SO_4_), hydrochloric acid (HCl) and phosphoric acid (H_3_PO_4_) were bought from Mojallali Chemical Co. Graphite (cat #332461), europium(iii) nitrate pentahydrate (Eu(NO_3_)_3_·5H_2_O), ammonium hydroxide (NH_4_OH), hydrazine hydrate (N_2_H_4_) and poly(tetra fluoro ethylene) (PTFE) were acquired from Sigma-Aldrich Co. Acetylene black (>99.9%, S. A. 80 m^2^ g^−1^) was procured from Alfa Aesar Co. The remaining materials were obtained from Merck Chemical Co.

### 2.2. Preparing EuNRs and EuNR-RGO

For the preparation of Eu_2_O_3_ nanorods, 10 mL of 0.1 M Eu^3+^ was combined with 15 mL deionized water (DW) into a 120 mL Teflon jar. Ammonia (28%) was added gradually until a white precipitate was formed. Then, the Teflon jar was sealed in a stainless-steel autoclave and heated in an oven for 12 h at 120 °C. The final product was washed repeatedly with DW and ethanol (96%), dried at 90 °C, and then calcined at 550 °C to obtain EuNRs.^33^

The hybrid EuNR-RGO was prepared *via* a sonochemical procedure (in an ultrasonic bath) through self-assembly, as reported in a previous work.^14^ Briefly, our procedure commonly included the synthesis of EuNRs and then placing the EuNRs on GO sheets, eventually reducing the loaded GO to RGO.

Six mg of the prepared EuNR powder was placed in an ultrasonic bath for 44 min to completely disperse it in 10 mL deionized water to obtain a suspension, which was continuously added to a 20 mL suspension of GO (3 mg GO in 10 mL DI water). The resulting mixture was placed under ultrasound vibration for 22 min. This was intended to anchor the nanorods onto the GO sheets. Finally, GO was reduced to RGO by adding a reducing agent (N_2_H_4_) in a boiling water bath. As a result, a black precipitate was formed and dried at 60 °C for 24 hours. Throughout the text, the material formed under these conditions shall be referred to as EuNR-RGO. Both pure EuNRs and RGO were prepared *via* the same method (*i.e.*, GO was prepared from graphite flake powder using the Tour's method^34^).

### 2.3. Characterizations

The crystallographic characterization was performed *via* X-ray diffraction (XRD) utilizing a Philips PW-1730 X-ray diffractometer equipped with Cu K_α_ radiation (*λ* = 1.5405 Å). The morphology of the materials was studied by field-emission scanning electron microscopy (FE-SEM) by means of MIRA3 TESCAN with a gold coating. Fourier transform infrared (FTIR) spectroscopy was performed using a BRUKER EQUINOX 55 spectrophotometer.

### 2.4. Electrochemical studies

**Fig. 1:**
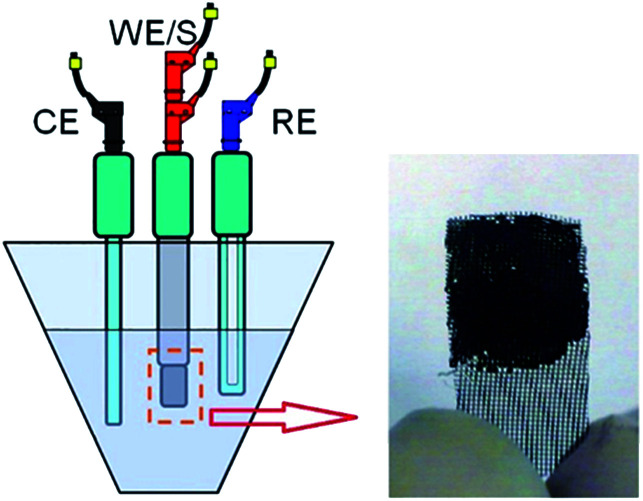
Schematic of a three-electrode system and an electrode sample made of steel grid.

In this research work, an electrochemical workstation (PGSTAT30, Auto lab, Netherlands) was applied and three-electrode electrochemical studies were carried out in a 3 M KOH aqueous solution. The working electrodes were fabricated for the electrochemical measurements by a mixture of the synthesized samples (*i.e.*, pure RGO and EuNRs and also EuNR-RGO nanocomposites) with carbon black, graphite and polytetrafluoroethylene at a 65 : 10 : 20 : 5 mass ratio and then dispersed in ethanol. In order to distribute the suspension over a current collector, a piece of rustproof steel, about 1 mg of the electro-active material and a 1 cm^2^ current collector were utilized for this purpose. Finally, the electrodes were dried in a vacuum oven at 80 °C for 4 h (Fig. 1). The electrodes constructed under these conditions have been referred to as EuNR-RGO throughout the text. Ag/AgCl and platinum electrodes were applied as the reference and counter electrodes, respectively. A three-electrode cell system was used and by electrochemical impedance spectroscopy (EIS), cyclic voltammetry (CV) and continuous cyclic voltammetry (CCV), the electrochemical performance was examined. To obtain CCV measurements, a home-made set was utilized, as described above.^14,35^

## 3. Results and discussion

### 3.1. Nanocomposite preparation

The preparation processes of hybrid materials made of graphene are classified into two major approaches: *in situ* and self-assembly processes. The *in situ* process involves the nucleation and growth of nanostructures in the presence of graphene derivatives (such as GO) in the reaction medium, but the other approach comprises mixing and self-assembly between previously formed nanostructures and graphene derivatives.

**Scheme 1:**
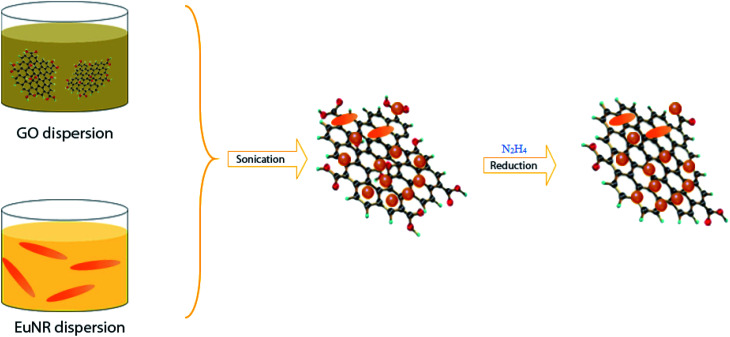
Schematic of the EuNR-RGO nanocomposite preparation process.

The latter one was actually used in this work (Scheme 1). EuNRs were prepared through the hydrothermal method and redistributed on GO by the sonochemical method. The FE-SEM image (Fig. 3b) implies that the nanorods comprise primary nanoparticles through an oriented attachment growth mechanism.^36^ Sonochemical phenomena (*i.e.*, high pressure and high-speed liquid jets caused by exploding bubbles) together with bonding between the europium oxide moieties and functional groups on GO caused the detachment of the EuNR moieties from rods to nanoparticles (Fig. 3) during the self-assembly process.^37^

### 3.2. Nanocomposite characterization

#### 3.2.1. XRD analysis

**Fig. 2:**
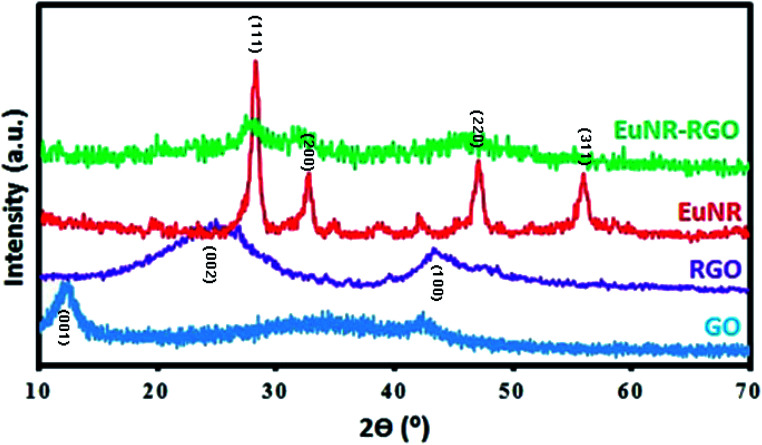
X-ray diffraction patterns of GO, RGO, EuNRs, and EuNR-RGO nanocomposites.

The XRD pattern of GO (Fig. 2a) shows a diffraction peak at 2*θ* = 12.2°, which is the obvious diffraction peak assigned to the (001) reflection of graphite oxide.^38^ However, because of the good reduction of GO to RGO, this peak disappeared in the XRD pattern of RGO.^38^ The XRD pattern of RGO shows two diffraction peaks at around 25° and 43°, which are attributed to the (002) and (100) planes of the graphite-like structure, respectively (Fig. 2b).^39^Fig. 2c shows the XRD pattern of the as-prepared EuNRs. This pattern demonstrates the cubic phase, which is in agreement with a previous report.^40^ The XRD pattern of EuNR-RGO (Fig. 2d) contains broad peaks, which indicate low crystallinity owing to the fact that through ultrasonic effects, re-dissolution of the nanorods on the surface of GO occurs to gain lower energy.^37^

#### 3.2.2. FE-SEM studies

**Fig. 3:**
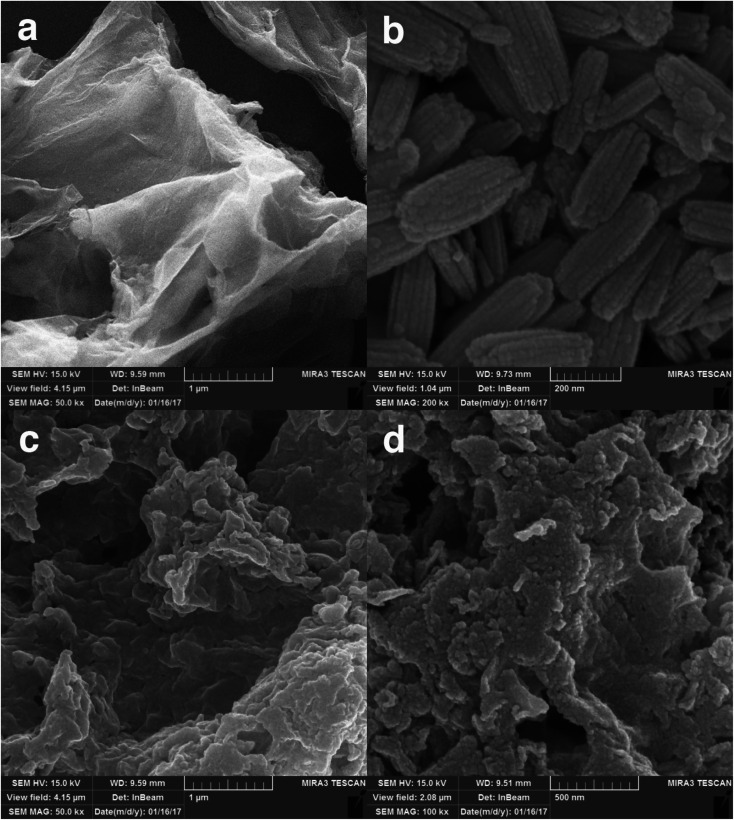
FE-SEM images of (a) GO, (b) EuNRs and (c and d) EuNR-RGO.

The layered structure of GO is illustrated in Fig. 3a. Fig. 3b shows the FE-SEM image of EuNRs. The diameter of these nanorods is about 30 nm. With a little attention, we can realize that these nanorods are interconnected along their length and have about 100 nm diameter. Actually, for the nanoparticles due to the high surface-to-volume ratio, high energy is obtained by increasing the above-mentioned parameter, so as to achieve a lower energy level. These nanoparticles should be altered to nanorod structures. Fig. 3c and d display the FE-SEM images of the EuNR-RGO nanocomposite, in which the distribution of EuNRs on RGO is observable. According to the figures, the combination between RGO and EuNRs is discernible. We observed a change in the morphology of EuNRs in pure and composite forms although the EuNRs underwent interconnection along their length in the pure form; this indicated that the formation of the nanocomposites prevented the accumulation of EuNRs and also, the nanorods prohibited the restacking of the RGO sheets. It could be seen that the nanorod structure of EuNRs vanished. This might be due to the re-dissolution of the nanorods caused by ultrasound effects. Ultrasonic irradiation causes a short duration of extremely high temperatures and pressures. Under these conditions and in the presence of GO, EuNRs dissolved and anchored on the surface of GO to achieve lower energy.^37^

#### 3.2.3. FT-IR study

**Fig. 4:**
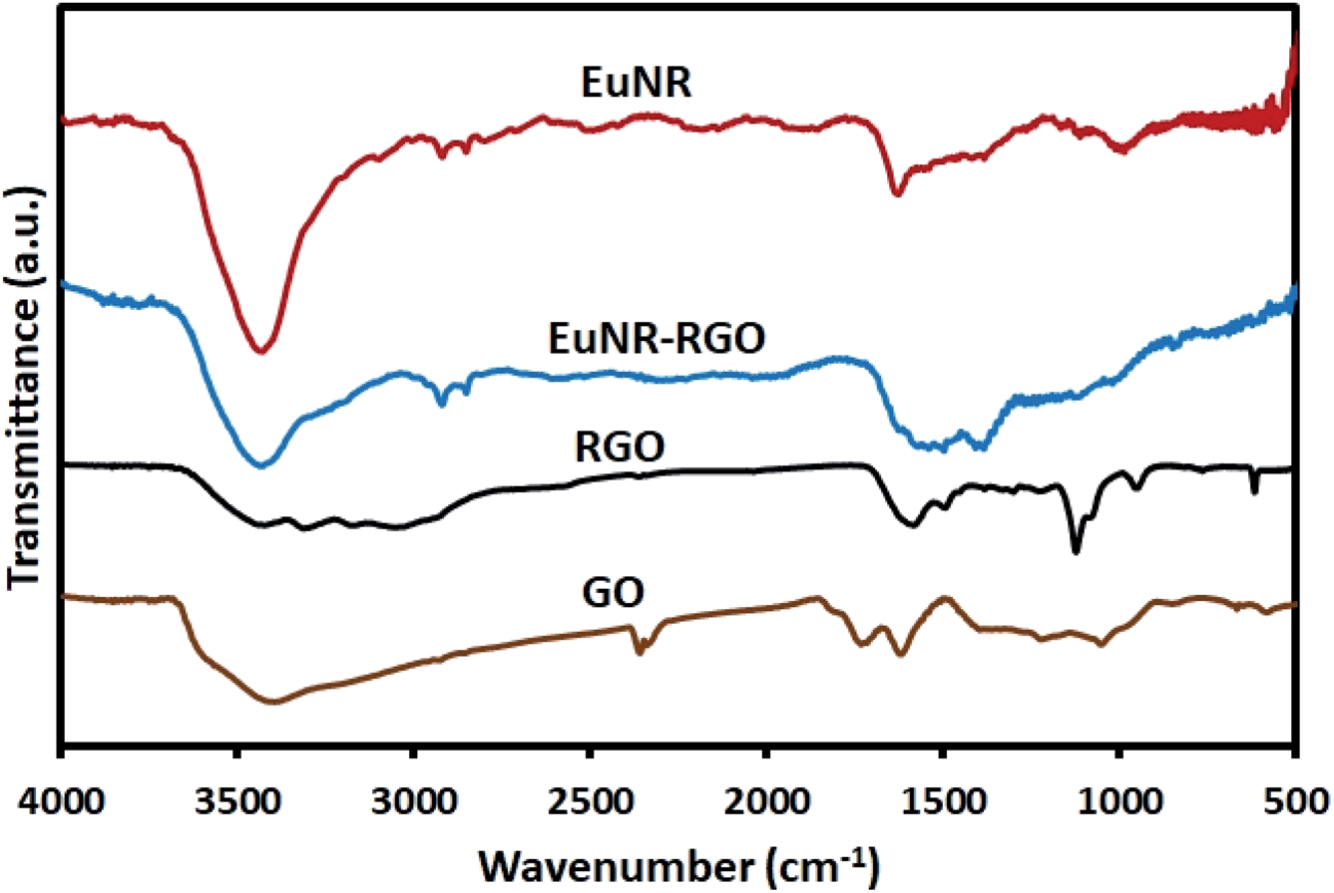
FT-IR spectra of EuNRs, EuNR-RGO nanocomposite, GO and RGO.

The FT-IR spectra of GO, RGO, EuNRs and EuNR-RGO are shown in Fig. 4. The FT-IR spectrum of GO indicates that GO is full of oxygen-containing functional groups such as carbonyl moieties (the C=O stretching vibration at 1735 cm^−1^), hydroxyl groups (the peak at 1054 cm^−1^), epoxy groups (the C–O asymmetric vibration at 1225 cm^−1^) and hydrogen bonding (the broad band of O–H stretching at around 3400 cm^−1^).^41^ Meanwhile, the peak at 1625 cm^−1^ attributed to aromatic C=C^42^ shifted dramatically to 1585 cm^−1^ after reduction in the spectra of both RGO and EuNR-RGO. It has been demonstrated that anchoring metal oxide nanostructures to the oxygen-containing functional groups such as epoxy, hydroxyl, carbonyl and carboxyl groups on the surface of GO has an important role in stacking them onto the sheets of graphene.^43^ Some of these functional groups (carboxyl and hydroxyl groups through the C–OH bonds) interact with metal oxides and the remaining functional groups are reduced, which causes a dramatic difference in the FT-IR spectra. Such an example is the peak at 980 cm^−1^ in the spectrum of EuNRs, which is assigned to the metal–oxygen bond;^44^ it shifted and reduced in intensity for the EuNR-RGO nanocomposite after anchoring nanoparticles to the surface functional groups of GO. Due to the reduction of GO by hydrazine at a high temperature, the characteristic absorption band of GO at 1735 cm^−1^ vanished, implying the successful reduction of GO in RGO and EuNR-RGO nanocomposite.^42^ Meanwhile, the peaks at 1088 cm^−1^ and 1124 cm^−1^ observed in the spectrum of RGO (and weakly in EuNR-RGO) are attributed to the C–N stretching vibration.^45^ The peak at 1384 cm^−1^ in the spectra of EuNR and EuNR-RGO is assigned to the adsorbed nitrosyl (N–O)^−^.^22^ The peak at 1545 cm^−1^ in the spectrum of EuNRs is related to the asymmetric vibration of the Eu–O bond. In addition, the peak at 1627 cm^−1^ in the spectra of EuNR and EuNR-RGO can be due to the bending vibration of water molecules.^46^

### 3.3. Electrochemical studies

#### 3.3.1. CV and specific capacitances

**Fig. 5:**
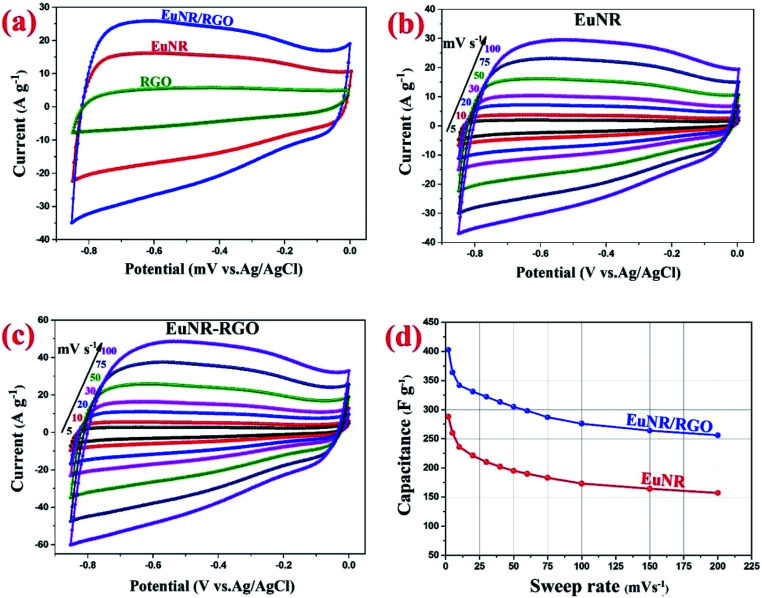
(a) Cyclic voltammograms of EuNR, RGO and EuNR-RGO electrodes in 3.0 M KCl at 50 mV s^−1^; (b) cyclic voltammograms of EuNR electrode in 3.0 M KCl at different scan rates from 5 to 100 mV s^−1^; (c) cyclic voltammograms of EuNR-RGO electrode in 3.0 M KCl at different scan rates from 5 to 100; and (d) capacitance *versus* sweep rate for EuNR and EuNR-RGO.

A 3-electrode system and 3 M KOH solution as the electrolyte were utilized for investigating the supercapacitive performance of the RGO, pure EuNR, and EuNR-RGO nanocomposite electrodes. The recorded CV curves were further used for the evaluation of the current responses of the materials under the mentioned conditions. Fig. 5 shows the results for the EuNR and EuNR-RGO electrodes, based on which the corresponding specific capacitance (SC) values were calculated using the following equation:^1^
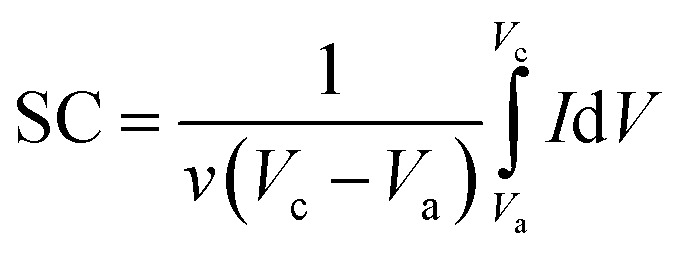
Here, *ν* (mV s^−1^) is the potential scan rate and the potential range determined with *V*_c_ and *V*_a_; the mass of the electroactive material was utilized for marking the response current (mA g^−1^).

Fig. 5a illustrates the cyclic voltammograms of RGO, pure EuNR and EuNR-RGO electrodes in 3.0 M KCl solutions at a scan rate of 50 mV s^−1^. The rectangular shapes of these curves confirm the high reversibility of the samples in this range and ideal pseudo-capacitive behavior.^1^ The larger rectangular area of EuNR-RGO implies the superior capacitance performance. The SC values at the scan rate of 50 mV s^−1^ were 76 F g^−1^, 195 F g^−1^ and 305 F g^−1^ for RGO, EuNR, and EuNR-RGO, respectively. The higher value for EuNR-RGO comes from the huge enhancement in the number of available active sites due to the synergism between the small particles of dispersed Eu_2_O_3_ and the preserved high surface area of RGO sheets.^47^Fig. 5b and c display the CV curves of EuNR and EuNR-RGO at various scan rates. The voltammograms are almost symmetric regarding the zero-current line due to the EDLC nature of the composite electrodes, showing a rapid faradaic reaction due to their pseudo-capacitive nature.

Ultimately, Fig. 5d shows SC *vs.* scan rate of EuNR and EuNR-RGO. By increasing the scan rate, the SC values of EuNR and EuNR-RGO decreased from 288 and 403 to 157 and 256 F g^−1^, respectively. Lower scan rates provide sufficient time for electrolyte ions such as K^+^ or H^+^ to diffuse into the internal electrode material pores, which causes an increase in the available surface for faradaic reactions, whereas higher scan rates limit this diffusion and hence decrease SC.^14,48^

#### 3.3.2. CCV

**Fig. 6:**
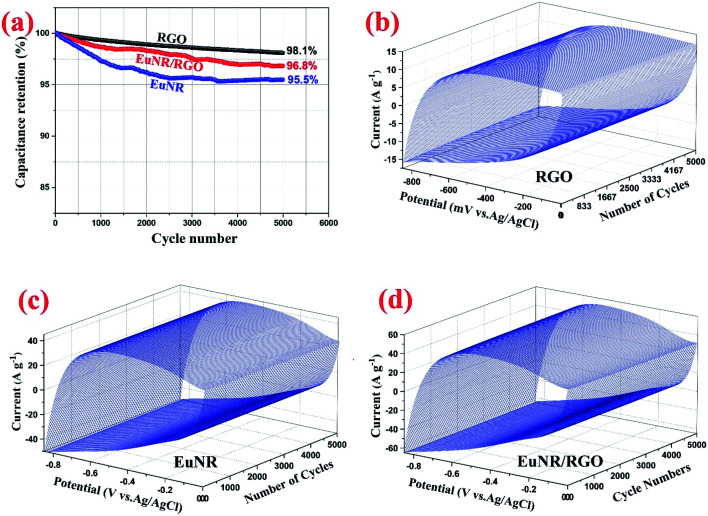
(a) RGO, EuNR and EuNR-RGO specific capacitance changes at 150 mV s^−1^. 3D-CCV curves at 150 mV s^−1^ for (b) RGO, (c) EuNR and (d) EuNR-RGO.

CCV experiments were carried out at 150 mV s^−1^ for 5000 cycles to evaluate the long-term cycle stability of EuNR and EuNR-RGO.^14^ As shown in Fig. 6a, the RGO, EuNR, and EuNR-RGO electrodes lost 1.9%, 4.5% and 3.2%, respectively, of their initial SC after 5000 cycles. The highest loss belongs to the EuNR electrode and the least loss is for RGO, which implies that the RGO content of the electrode is significantly crucial for cycle stability. Fig. 6b–d show three-dimensional (3D) CCVs conducted at a scan rate of 150 mV s^−1^ during 5000 cycles, which clearly illustrate the stability of voltammograms over a large number of cycles.

#### 3.3.3. Galvanostatic charge/discharge

**Fig. 7:**
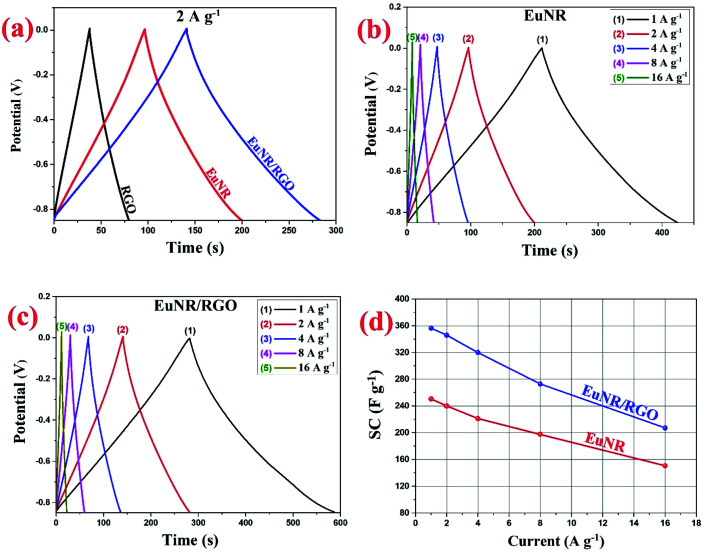
(a) RGO, EuNR and EuNR-RGO charge/discharge curves at 2.0 A g^−1^ current density; (b) EuNR charge/discharge curves at 1–16 A g^−1^ current densities; (c) EuNR-RGO charge/discharge curves at 1–16 A g^−1^ current densities and (d) EuNR and EuNR-RGO specific capacitance changes with changing current densities from 1 to 16 A g^−1^.

To appraise the electrodes' supercapacitive performance, charge/discharge tests were conducted using a two-electrode system. Curves for the RGO, EuNR and EuNR-RGO electrodes were obtained at a current density of 2.0 A g^−1^ (Fig. 7a). All the curves have symmetric sharp equilateral triangular shapes, which imply ideal capacitor performance. Correspondingly, the following equation^1^ was utilized to calculate the values of SC:
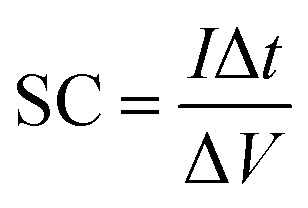


In this equation, the discharge time (s), charge/discharge current (A) and potential drop (V) are represented by Δ*t*, *I* and *V*, respectively.

In the case of EuNR-RGO, a particular capacitance of 345.9 F g^−1^ was achieved at 2 A g^−1^, which is the largest value among all other electrodes. Fig. 7b and c display the charge/discharge curves from 1 to 16 A g^−1^ current densities in the range from 0.1 to −0.9 V for both EuNR and EuNR-RGO. In both cases, the shapes of the curves are similar to an equilateral triangle, which implies reversibility and ideal capacitive behavior during the charge/discharge processes. Significant electrical conductivity raised from RGO and facilitated redox reactions due to the small size of Eu_2_O_3_ together with the accelerated charge transport achieved from the synergy between RGO and EuNR would be the reasons for the superior capacitive behavior of EuNR-RGO.^49^

**Fig. 8:**
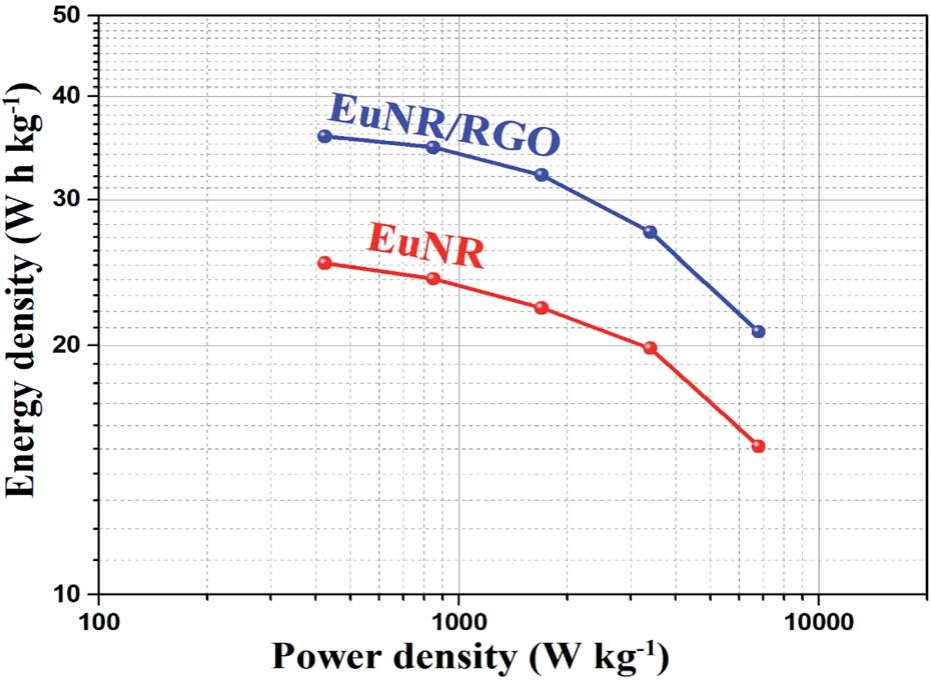
Ragone plots of EuNR and EuNR-RGO.

Fig. 8 illustrates the superior performance of EuNR-RGO in comparison with the other electrodes, which was also confirmed by Ragone plots. The charge/discharge analyses at diverse current densities were used to calculate energy and power densities, which are necessary for this plot. At a power density of 425 W kg^−1^, the maximum energy density (*i.e.*, 35.8 W h kg^−1^) was obtained for EuNR-RGO. In comparison to the other electrodes reported in literature, the gained value was very high.^50–52^ According to the energy and power densities, EuNR-RGO was considered as a suitable material for supercapacitor electrodes.

#### 3.3.4. EIS studies

**Fig. 9:**
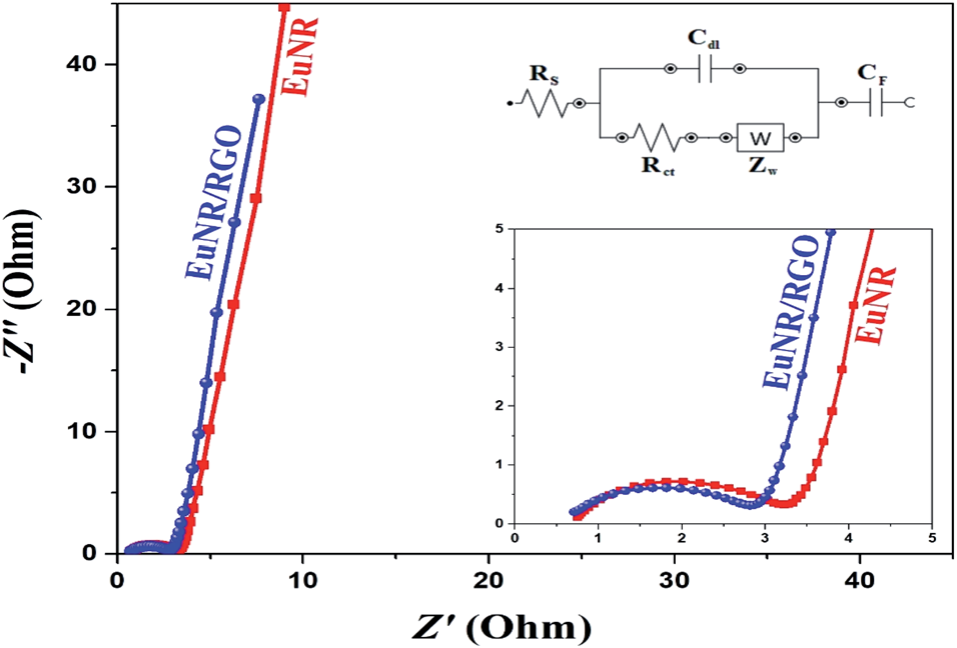
Impedance spectra of EuNR and EuNR-RGO. The frequency range is 0.1–10^5^ Hz.

EIS is an imperative technique to evaluate and compare the basic performance of materials for their application as supercapacitor electrodes. The EIS data of EuNR and EuNR-RGO obtained in the range of 0.1–10^5^ Hz are shown in Fig. 9. In the high-frequency region of the Nyquist plot, both of them caused an arc due to charge transfer through the electrolyte and electrode. At lower frequencies, owing to the diffusion of ions into the electrode, this is followed by a tail. On the other hand, the electrochemical reaction impedance affects the size of the arc, indicating that a smaller arc radius corresponds to smaller charge transfer resistance.^53^ The EIS curves (Fig. 9) were examined for an equivalent circuit *via* the complex nonlinear least squares (CNLS) fitting method.^54^ The equivalent circuit contains five elements: *R*_s_, *R*_ct_, *C*_dl_, *Z*_W_, and *C*_F_. *R*_s_ is the internal resistance, which comprises the active materials' natural resistance, bulk electrolyte resistance and electrolyte ionic resistance at the interface between the electrode and current collector;^55^*Z*_W_ represents the Warburg resistance, which demonstrates that the ion diffusion/transport to the surface of the electrode is dependent on frequency;^56^*R*_ct_ is the resistance of the interfacial charge transfer between the electrode and electrolyte; *C*_dl_ is the capacitance due to the electrical double layer at the interface between the electrode and electrolyte; and *C*_F_ is the pseudo-capacitance of the faradaic reaction.

The equivalent circuit values are demonstrated in Table 1. It is clear that the value of *R*_ct_ for EuNR-RGO is less than others. When *R*_ct_ is less, it means that the electrochemical reaction is more facile at the electrode/electrolyte interface. Similarly, Table 1 illustrates that EuNR-RGO reveals enhanced electrochemical performance rather than EuNR. In addition, EuNR-RGO demonstrates more ideal Warburg resistance (more ideal capacitance behavior results in a more vertical line).^57^ As confirmed by chronopotentiograms and CVs, EIS further reveals synergism between EuNRs and RGO.

**Table 1:** Values determined by CNLS fitting

	EuNR	EuNR-RGO
*R*_s_ (ohm)	0.75	0.71
*C*_dl_ (mF)	2.75	2.12
*R*_ct_ (ohm)	2.61	2.19
*Z*_W_ (mMho)	540	603
*C*_F_ (mF)	445	479

## 4. Conclusion

EuNR-RGO nanocomposites were synthesized by a facile sonochemical method, and the measurements indicated that the presence of RGO caused better conductivity by reducing the ionic mass-transfer resistance. Experiments proved that the SC, stability and energy density of the EuNR-RGO nanocomposite electrodes were superior to those of the electrodes based on pure RGO or Eu_2_O_3_. As a result, a composition of 1 : 1 wt of Eu_2_O_3_ nanorods to RGO caused the extraordinary supercapacitive behavior with an SC of about 403 F g^−1^ at a scan rate of 2 mV s^−1^. This was extremely superior in comparison to those of both the RGO and Eu_2_O_3_ nanorods. The CCV technique at 150 mV s^−1^ was used to investigate the nanocomposite electrode's stability. The SC of EuNR-RGO remained at almost 96.8% of its initial value after 5000 cycles. These data were further confirmed by EIS and galvanostatic charge/discharge. Therefore, the optimal EuNR-RGO nanocomposites are suitable materials for the construction of high-performance supercapacitor electrodes.

## Conflicts of interest

There are no conflicts to declare.

## Supplementary Material

## References

[cit1] Shiralizadeh Dezfuli A., Kohan E., Naderi H. R., Salehi E. (2019). New J. Chem..

[cit2] Wu Z. S., Zhou G., Yin L. C., Ren W., Li F., Cheng H. M. (2012). Nano Energy.

[cit3] Jahng Y., Kwon O. K., Lee S. (2012). Arch. Pharmacal Res..

[cit4] Wang Y., Guo C. X., Liu J., Chen T., Yang H., Li C. M. (2011). Dalton Trans..

[cit5] Naderi H. R., Mortaheb H. R., Zolfaghari A. (2014). J. Electroanal. Chem..

[cit6] Pandolfo A. G., Hollenkamp A. F. (2006). J. Power Sources.

[cit7] Wang G., Zhang L., Zhang J. (2012). Chem. Soc. Rev..

[cit8] Joung D., Singh V., Park S., Schulte A., Seal S., Khondaker S. I. (2011). J. Phys. Chem. C.

[cit9] Liu B., Jiang L., Yao M., Liu B., Li Q., Liu R., Lv H., Lu S., Gong C., Zou B., Cui T., Hu G., Wågberg T. (2012). J. Phys. Chem. C.

[cit10] Yu S., Liu Q., Yang W., Han K., Wang Z., Zhu H. (2013). Electrochim. Acta.

[cit11] Teymourian H., Salimi A., Khezrian S. (2013). Biosens. Bioelectron..

[cit12] Ji Z., Shen X., Li M., Zhou H., Zhu G., Chen K. (2013). Nanotechnology.

[cit13] Naderi H. R., Ganjali M. R., Dezfuli A. S., Norouzi P. (2016). RSC Adv..

[cit14] Dezfuli A. S., Ganjali M. R., Naderi H. R., Norouzi P. (2015). RSC Adv..

[cit15] Rakhi R. B., Chen W., Cha D., Alshareef H. N. (2011). J. Mater. Chem..

[cit16] Liao Q., Li N., Jin S., Yang G., Wang C. (2015). ACS Nano.

[cit17] He W., Lin J., Wang B., Tuo S., Pantelides S. T., Dickerson J. H. (2012). Phys. Chem. Chem. Phys..

[cit18] Wang B., Park J., Wang C., Ahn H., Wang G. (2010). Electrochim. Acta.

[cit19] Adachi G. , Imanaka N. and Kang Z. C., Binary rare earth oxides, Springer Netherlands, 2005

[cit20] Rosynek M. P. (1977). Catal. Rev..

[cit21] Jhang J. H., Schaefer A., Cartas W., Epuri S., Bäumer M., Weaver J. F. (2013). J. Phys. Chem. C.

[cit22] Tsujimoto S., Masui T., Imanaka N. (2015). Eur. J. Inorg. Chem..

[cit23] Antolini E., Perez J. (2011). Int. J. Hydrogen Energy.

[cit24] Kohan E., Shiralizadeh Dezfuli A. (2019). J. Mater. Sci.: Mater. Electron..

[cit25] Johnson D. A. (1980). J. Chem. Educ..

[cit26] Jafari H., Ganjali M. R., Shiralizadeh Dezfuli A., Kohan E. (2018). J. Mater. Sci.: Mater. Electron..

[cit27] Song X. C., Yang E., Ma R., Chen H. F., Ye Z. L., Luo M. (2009). Appl. Phys. A: Mater. Sci. Process..

[cit28] Pol V. G., Palchik O., Gedanken A., Felner I. (2002). J. Phys. Chem. B.

[cit29] Du N., Zhang H., Chen B., Wu J., Li D., Yang D. (2007). Nanotechnology.

[cit30] Wang X., Li Y. (2003). Chem.–Eur. J..

[cit31] Zhang L., Jiu H., Luo J., Chen Q. (2007). J. Cryst. Growth.

[cit32] Qian L., Gui Y., Guo S., Gong Q., Qian X. (2009). J. Phys. Chem. Solids.

[cit33] Kang J. G., Jung Y., Min B. K., Sohn Y. (2014). Appl. Surf. Sci..

[cit34] Marcano D. C., Kosynkin D. V., Berlin J. M., Sinitskii A., Sun Z., Slesarev A., Alemany L. B., Lu W., Tour J. M. (2010). ACS Nano.

[cit35] Norouzi P., Garakani T. M., Ganjali M. R. (2012). Electrochim. Acta.

[cit36] Shiralizadeh Dezfuli A., Ganjali M. R., Norouzi P. (2014). Mater. Sci. Eng., C.

[cit37] Dezfuli A. S., Ganjali M. R., Norouzi P., Faridbod F. (2015). J. Mater. Chem. B.

[cit38] Liu B., Jiang L., Yao M., Liu B., Li Q., Liu R., Yao Z., Lu S., Cui W., Hua X., Zou B., Cui T. (2013). CrystEngComm.

[cit39] Huang K., Lei M., Wang Y. J., Liang C., Ye C. X., Zhao X. S., Li Y. F., Zhang R., Fan D. Y., Wang Y. G. (2014). Powder Diffr..

[cit40] Yan T., Zhang D., Shi L., Li H. (2009). J. Alloys Compd..

[cit41] Dezfuli A. S., Ganjali M. R., Jafari H., Faridbod F. (2017). J. Mater. Sci.: Mater. Electron..

[cit42] Ren P. G., Yan D. X., Ji X., Chen T., Li Z. M. (2011). Nanotechnology.

[cit43] Jastrzębska A. M., Karcz J., Letmanowski R., Zabost D., Ciecierska E., Zdunek J., Karwowska E., Siekierski M., Olszyna A., Kunicki A. (2016). Appl. Surf. Sci..

[cit44] Belokopytov Y. V., Kholyavenko K. M., V Gerei S. (1979). J. Catal..

[cit45] Liu H., Hu X., Guo H., Zhao J., Li F., Zhu D., Liu S. (2019). Phys. Chem. Chem. Phys..

[cit46] Jafari H., Ganjali M. R., Shiralizadeh Dezfuli A., Kohan E. (2018). J. Mater. Sci.: Mater. Electron..

[cit47] jing Yang Y., Liu E. H., min Li L., zheng Huang Z., jie Shen H., xia Xiang X. (2009). J. Alloys Compd..

[cit48] Jafari H., Ganjali M. R., Dezfuli A. S., Faridbod F. (2018). Appl. Surf. Sci..

[cit49] Choi H. J., Jung S. M., Seo J. M., Chang D. W., Dai L., Baek J. B. (2012). Nano Energy.

[cit50] Li B., Fu Y., Xia H., Wang X. (2014). Mater. Lett..

[cit51] Zhang X., Sun X., Zhang H., Zhang D., Ma Y. (2012). Mater. Chem. Phys..

[cit52] Yu G., Hu L., Vosgueritchian M., Wang H., Xie X., McDonough J. R., Cui X., Cui Y., Bao Z. (2011). Nano Lett..

[cit53] Wang G., Zhang J., Kuang S., Zhou J., Xing W., Zhuo S. (2015). Electrochim. Acta.

[cit54] Conway B. E. , Electrochemical Supercapacitors, Springer US, 1st edn, 1999

[cit55] Zolfaghari A., Naderi H. R., Mortaheb H. R. (2013). J. Electroanal. Chem..

[cit56] Zhang K., Zhang L. L., Zhao X. S., Wu J. (2010). Chem. Mater..

[cit57] Li L., Seng K. H., Liu H., Nevirkovets I. P., Guo Z. (2013). Electrochim. Acta.

